# Late Health Effects of Partial Body Irradiation Injury in a Minipig Model Are Associated with Changes in Systemic and Cardiac IGF-1 Signaling

**DOI:** 10.3390/ijms22063286

**Published:** 2021-03-23

**Authors:** Bernadette Hritzo, Saeed Y. Aghdam, Betre Legesse, Amandeep Kaur, Maohua Cao, Marjan Boerma, Nabarun Chakraborty, George Dimitrov, Aarti Gautam, Rasha Hammamieh, William Wilkins, Alena Tsioplaya, Gregory P. Holmes-Hampton, Maria Moroni

**Affiliations:** 1 Armed Forces Radiobiology Research Institute, Bethesda, MD 20889, USA; bernadette.hritzo.ctr@usuhs.edu (B.H.); saeed.aghdam.ctr@usuhs.edu (S.Y.A.); betre.legesse.ctr@usuhs.edu (B.L.); noor4aman@yahoo.com (A.K.); wlouiswilkins@gmail.com (W.W.); alena.tsioplaya@usuhs.edu (A.T.); gregory.holmes-hampton@usuhs.edu (G.P.H.-H.); 2 Department of Endodontics, Texas A&M College of Dentistry, Dallas, TX 75246, USA; maohuacao@gmail.com; 3 Department of Pharmaceutical Sciences, University of Arkansas for Medical Sciences (UAMS), Little Rock, AR 72205, USA; MBoerma@uams.edu; 4 Medical Readiness Systems Biology, Walter Reed Army Institute of Research, Silver Spring, MD 20910, USA; nabarun.chakraborty2.civ@mail.mil (N.C.); george.i.dimitrov.ctr@mail.mil (G.D.); 5 The Geneva Foundation, Walter Reed Army Institute of Research, Silver Spring, MD 20910, USA; aarti.gautam.civ@mail.mil (A.G.); rasha.hammamieh1.civ@mail.mil (R.H.)

**Keywords:** radiation, late effects, Göttingen minipig, partial body irradiation, Insulin-like growth factor-1, IGF-1

## Abstract

Clinical, epidemiological, and experimental evidence demonstrate non-cancer, cardiovascular, and endocrine effects of ionizing radiation exposure including growth hormone deficiency, obesity, metabolic syndrome, diabetes, and hyperinsulinemia. Insulin-like growth factor-1 (IGF-1) signaling perturbations are implicated in development of cardiovascular disease and metabolic syndrome. The minipig is an emerging model for studying radiation effects given its high analogy to human anatomy and physiology. Here we use a minipig model to study late health effects of radiation by exposing male Göttingen minipigs to 1.9–2.0 Gy X-rays (lower limb tibias spared). Animals were monitored for 120 days following irradiation and blood counts, body weight, heart rate, clinical chemistry parameters, and circulating biomarkers were assessed longitudinally. Collagen deposition, histolopathology, IGF-1 signaling, and mRNA sequencing were evaluated in tissues. Our findings indicate a single exposure induced histopathological changes, attenuated circulating IGF-1, and disrupted cardiac IGF-1 signaling. Electrolytes, lipid profiles, liver and kidney markers, and heart rate and rhythm were also affected. In the heart, collagen deposition was significantly increased and transforming growth factor beta-1 (TGF-beta-1) was induced following irradiation; collagen deposition and fibrosis were also observed in the kidney of irradiated animals. Our findings show Göttingen minipigs are a suitable large animal model to study long-term effects of radiation exposure and radiation-induced inhibition of IGF-1 signaling may play a role in development of late organ injuries.

## 1. Introduction

Clinical, epidemiological, and experimental evidence clearly indicate the existence of non-cancer effects of ionizing radiation. Data from survivors of the Hiroshima and Nagasaki bombings [1], from cancer patients exposed to radiotherapy at young ages [2], and from the 30-year follow up on Ukrainian workers involved in the Chernobyl accident [3] suggest the leading causes of late deaths from radiation exposure are heart disease and stroke, and that radiation exposure has a profound effect on the endocrine system via hormonal imbalances in exposed subjects [4].

Deficiency in growth hormones, obesity, and metabolic disorders including dyslipidemia, metabolic syndrome, diabetes, and hyperinsulinemia are commonly observed in surviving pediatric cancer patients who received radiotherapy with the pituitary-hypothalamic regions situated in the field of irradiation [5,6]. A two-fold increase in the incidence of type-2 diabetes has been documented among survivors of the Hiroshima bomb [7] and an increased incidence in type-1 diabetes and imbalances in blood insulin levels have been reported in individuals exposed to higher radiation levels in Belarus between 1986 and 1999 following the Chernobyl accident [8,9]. Preclinical studies in primates have confirmed the link between radiation exposure and increased incidence of type-2 diabetes in irradiated monkeys, 5–9 years after radiation exposure [10].

In addition to the endocrine system, the cardiovascular system has also been demonstrated to be more radio-sensitive than previously recognized [11,12]. In breast cancer patients, the risk of ischemic heart diseases has been shown to increase after exposure to doses ranging from 1 to 5 Gy [13]. Among atomic bomb survivors, exposure to doses 0.5 Gy and above were associated with an increased risk of cardiovascular disease later in life [14,15]. Radiation-induced cardiovascular diseases include heart failure, valvular heart disease, coronary artery disease, constrictive pericarditis, restrictive cardiomyopathy, infiltration of inflammatory cells, collagen deposition, fibrosis, cardiac conduction abnormalities, and associated arrhythmia [2,16,17].

Understanding the late health effects of radiation exposure is required for the identification of targets for medical intervention following clinical or accidental exposure to survivable or environmental doses of radiation. Consistent evidence from in vitro and in vivo studies demonstrate endocrine disorders, metabolic imbalance, and cardiovascular defects are all characterized by imbalance in the Insulin-like growth factor-1 (IGF-1) hormone and its affiliated signaling [18,19,20,21,22,23,24,25,26,27,28]. This indicates IGF-1 plays a major role in the pathophysiology of both cardiovascular disease and metabolic syndrome and, therefore, potentially in radiation-induced early or late health effects [18,19,20,21,29].

The small anabolic hormone, IGF-1, is produced primarily by the liver and is regulated by growth hormone (GH). It modulates muscle and bone growth, organ health and development, and insulin activity, thus orchestrating protein and lipid metabolism and regulating oxidative stress [19,22,23,24,30]. IGF-1 is critical for heart structure and function and acts as a cardioprotector by enhancing insulin sensitivity and tissue glucose utilization, therefore promoting heart contractility and regulating cardio-vascular homeostasis [23,25]. A prospective nested case–control study within the larger Dan-MONICA study (The Denmark-Glostrup population for the MONItoring trends in CArdiovascular diseases study) on cardiovascular epidemiology indicated otherwise healthy participants with initially low levels of circulating IGF-1 had a significantly higher risk of developing ischemic heart disease at the 15-year clinical follow up [26]. Similar results were observed in a longitudinal study among Laron dwarfs in Ecuador [27]. In this population, which is characterized by severe congenital IGF-1 deficiency, low IGF-1 levels were associated with a shorter life span, cardiac atrophy, and impaired cardiac performance. Almost half of the individuals in the study died from stroke and cardiovascular diseases. Likewise, in the general population, IGF-1 deficiency has also been associated with increased risk for cardiovascular and cerebrovascular diseases, coronary heart and artery disease, ischemic heart disease, ischemic stroke, myocardial infarction, atherothrombotic diseases, impairment of contractility, arrhythmia, collagen deposition, endothelial dysfunction, oxidative stress, and chronic low-grade inflammation [27].

The critical role of IGF-1 in cardiac health has been confirmed using several genetically engineered rodent models of IGF-1 deficiency [27]. Lewis dwarf rats are characterized by GH/IGF-1–deficiency presenting with pathophysiological alterations also observed in human GH/IGF-1 deficient patients including decreased myocyte size, cardiac atrophy, impaired cardiac contractility and diastolic function, and reduction in the ability of the myofilaments to respond to calcium [31,32]. Ames and Snell dwarf mice are characterized by IGF-1 deficiency in development which results in dwarfism, altered metabolism, reduced myocyte size, cardiac atrophy, and deregulated cardiac contractility profiles [28]. The Little mouse, likewise characterized by low IGF-1 circulating levels, displays cardiac atrophy and impaired systolic and diastolic function [33]. Impairment of the IGF-1 pathway by either destruction of the IGF-1 receptor (IGF-1R) or overexpression of IGF-1 inhibitory proteins is associated with dysregulation of nuclear factor-like 2 (Nrf2)-dependent antioxidant responses, leading to increased vascular oxidative stress and endothelial dysfunction [34,35].

Besides its roles in cardiac physiology, IGF-1 is involved in the development and function of most major organs [36]. In the kidney, it regulates renal hemodynamics, enhances glomerular filtration rate, increases glomerular perfusion, and promotes renal reabsorption of sodium, phosphate, and calcium. Renal IGF-1 signaling deficiency decreases glomerular filtration rate, renal plasma flow and lowers sodium and water levels [37]. In the liver, IGF-1 signaling regulates hepatic architecture and gene expression (cytoskeleton, hepatocyte polarity, cell junctions, and extracellular matrix proteins), reduces oxidative stress, and improves mitochondrial function [38].

In addition to its ability to regulate organ development and function, IGF-1 plays important roles in the biological response of tissues to radiation exposure due to its ability to promote cell survival, DNA repair, and protection from apoptosis and oxidative stress [19,39]. The IGF-1 pathway is known to mediate resistance to radiotherapy in several types of cancers and has been suggested as a potential target to potentiate radiation therapy [40,41];perturbation of the insulin/IGF-1 phosphatidylinositiol 3-kinase (PI3K)-protein kinase B (Akt) signaling pathway in normal tissues has been associated with short- and long-term radiation-induced vascular dysfunction and death [21,42,43,44,45]. In particular, we have shown in a minipig model of the Acute Radiation Syndrome, IGF-1 and its downstream signaling cascade are altered at clinically relevant doses of radiation; deficit in IGF-1 signaling in the presence of systemic inflammation was associated with alterations in both the cardiovascular and metabolic systems and poor survival outcome within two months after irradiation [21,43,45]. The Göttingen minipig offers an advantageous model to study radiation effects given the similarity of minipig anatomical and physiological composition to that of humans [46,47,48]. While Göttingen minipigs are more radiosensitive than humans, we have previously established the minipig responds to radiation exposure similarly to humans and overlapping acute disease progression parameters are observed in both species [49,50].

Following the well-established relationship between congenital IGF-1 deficit and onset of cardiovascular and metabolic diseases in humans [18] along with the radiation-induced IGF-1 signaling deterioration observed in radiotherapy patients and in preclinical models of radiation injury [42,43,45], we attempted to begin establishing a large animal model to study the late effects of radiation, specifically one that could mimic IGF-1 deficiency and its associated development of cardio-metabolic abnormalities after exposure to relatively low doses of radiation. For this purpose, we exposed male Göttingen minipigs to partial body irradiation (PBI) (whole body X-ray with 10% bone marrow sparing) at clinically relevant radiation doses and evaluated the occurrence of late effects of radiation exposure after 120 days with particular focus on changes in IGF-1 signaling and onset of heart, kidney, and liver pathologies.

## 2. Results

### 2.1. Partial Body Irradiation Allowed for Recovery from Hematopoietic Cell Loss and Induction of Inflammation

**Figure 1:**
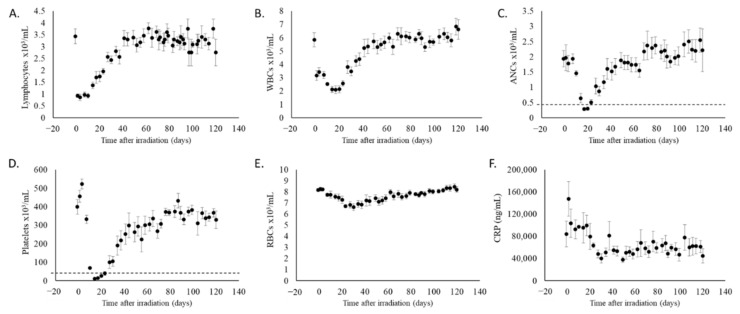
Evaluation of lymphocytes, white blood cells (WBCs), absolute neutrophil counts (ANCs), platelets, red blood cells (RBCs) and C-reactive protein (CRP) levels in irradiated animals. (**A**) Lymphocyte, (**B**) WBC, (**C**) ANC, (**D**) platelet, and (**E**) RBC counts were determined longitudinally in whole blood and CRP levels (**F**) were measured by ELISA in plasma collected throughout the study; values are reported as average +/− standard error of the mean (sem). Dashed line marks the threshold of severe cytopenia (0.5 × 10^3^/μL for neutrophils and 20 × 10^3^/μL for platelets).

To assess late effects of partial body irradiation exposure, eight adult Göttingen minipigs were exposed to either 1.9 or 2.0 Gy 4 MV LINAC -derived photons (Linear Accelerator, Elekta, Stockholm, Sweden) with 10% bone marrow sparing and monitored for 120 days. One animal exposed to 2 Gy succumbed to acute radiation-induced injuries 15 days after exposure; this animal was excluded from all analyses. The remaining animals were monitored daily for general clinical signs, metabolic effects, and hematological toxicity. Figure 1 panels A–E report the dynamics of hematopoietic cell loss and recovery. The bone marrow sparing of the PBI model allowed hematopoietic recovery and increased survivability at doses otherwise potentially lethal [45,49]. C-reactive protein (CRP) levels declined over time in animals with bone marrow sparing regardless of radiation dose received (Figure 1F) indicating the absence of acute systemic inflammation, unlike what is observed following total body irradiation [50].

### 2.2. Irradiation Slowed Weight Gain and Decreased Heart Rate

**Figure 2:**
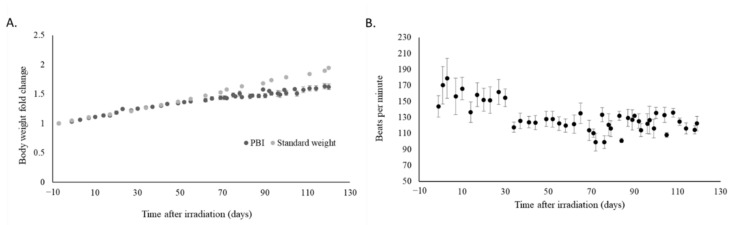
Evaluation of body weight and heart rate in irradiated animals. (**A**) Body weight and (**B**) heart rate were measured throughout the study in minipigs exposed to PBI; heart rates were reported as averages +/− sem (**B**). Body weight measurements were compared to values from reference age-matched, non-irradiated animals provided by the vendor and plotted as a control (standard weight).

Having seen recovery in the hematopoietic system and reduction of systemic inflammation, we evaluated whether partial body irradiation influenced measurable physiological parameters. No major changes were observed in terms of body temperature, respiration, and systolic and diastolic blood pressure throughout the study (not shown). The rate of body weight gain in irradiated animals was decreased as compared to the data provided by the vendor for the rate of weight gain in age-matched, non-irradiated pigs (Figure 2A). Heart rate declined over time (Figure 2B) and incidents of arrhythmia (as determined by auscultation of heart rhythm) occurred in all irradiated animals.

### 2.3. Organ Damage and Fibrosis Were Induced by Radiation

Histological analysis (hematoxylin and eosin (H&E)) revealed bone marrow hypocellularity, spleen lymphoid hypoplasia, and testicle degeneration in irradiated animals. Expert examination of heart (left ventricle), lung (right diaphragmatic lobe), liver and kidney (left) sections stained with Masson’s Trichrome determined the incidence of minimal to mild fibrosis in the kidney (100%) and lungs (43%) of irradiated animals; while fibrosis was not detected outright in the heart, arterial pigmentation was detected in three of the seven irradiated animals (Table 1). Pulmonary hemorrhage, congestion, and edema and renal nephropathy were also observed occasionally by H&E (Table 1) in the irradiated animals. Markers of atherosclerosis in the aorta were not seen in irradiated animals. Aside from the observation of a pulmonary granuloma in one control animal and an instance of pulmonary hemorrhage and congestion in another, no gross histological lesions among evaluated organs were noted in control animals.

### 2.4. Collagen Deposition Increased in Heart and Kidneys of Irradiated Animals 

**Figure 3:**
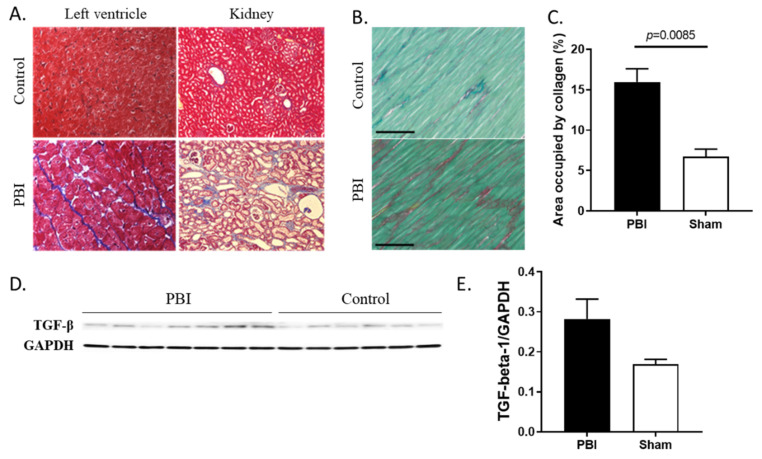
Analysis of fibrosis markers in heart and kidney samples of control and irradiated minipigs. (**A**) Histological analysis of collagen deposition in heart left ventricle (left) and kidney (right) sections using Masson’s Trichrome staining (blue staining). (**B**) Collagen deposition in the left ventricle was quantified using Sirius Red (red staining) supplemented with Fast Green to visualize cytoplasm; representative photomicrographs (20× magnification; 100 μm scale bar) are shown. (**C**) The percentage of left ventricular area occupied by collagen (field of view) was measured and averages are reported +/− sem (control: *n* = 3; PBI: *n* = 7). (**D**) TGF-beta-1 expression was assessed by western blot analysis in heart lysates of irradiated and control animals; Glyceraldehyde 3-phosphate dehydrogenase (GAPDH) was used as loading control. (**E**) Densitometric quantification was performed and values were normalized to GAPDH and presented as averages +/− sem.

Histological analysis was also used to determine if radiation triggered fibrosis in the heart and kidneys. Collagen deposition in the left ventricle was confirmed by Masson’s Trichrome staining (Figure 3A, left) and quantified by Sirius Red staining (Figure 3B,C). The collagen deposition in the irradiated animals was significantly (*p* = 0.0085) higher as compared to that seen in the controls (Figure 3C). In addition, presence of transforming growth factor beta-1 (TGF-beta-1), a cytokine involved in tissue repair and collagen deposition, was induced, although not significantly (*p* = 0.0648), in the heart of irradiated animals as shown by western blot analysis and densitometric quantification (Figure 3D,E). Additionally, collagen deposition was observed in the kidney sections stained with Masson’s Trichrome (Figure 3A, right).

### 2.5. Radiation Exposured Altered Plasma IGF-1 Levels, Lipid Profiles, and Liver and Kidney Function

**Figure 4:**
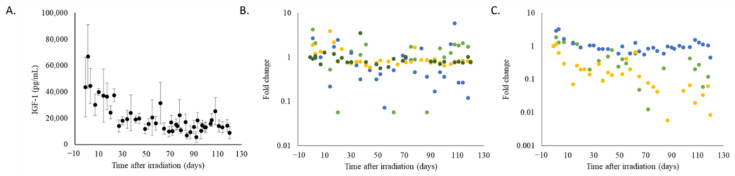
Analysis of plasma IGF-1 levels in irradiated animals by ELISA. (**A**) Values are shown as averages +/− sem over time. (**B**) Data from individual animals were normalized to pre-irradiation levels and grouped by radiation dose; 1.9 Gy (*n* = 4 animals, individual measurements for a given animal are represented by data points of a single color: dark green, blue, green, or yellow) and (**C**) 2.0 Gy (*n* = 3 animals, individual measurements for a given animal are represented by data points of a single color: blue, green, or yellow).

In irradiated animals, plasma levels of IGF-1, lipid profiles, and clinical chemistry parameters that reflect organ function were altered. Plasma IGF-1 levels declined over time in all irradiated animals (Figure 4A). However, attenuation of plasma IGF-1 was less severe in four irradiated animals; while the sample size is limited and dose-dependence for IGF-1 attenuation cannot be fully determined, three out of four animals irradiated at 1.9 Gy had less severe IGF-1 attenuation, whereas one out of three animals irradiated at 2.0 Gy had less severe IGF-1 attenuation (Figure 4B,C).

**Figure 5:**
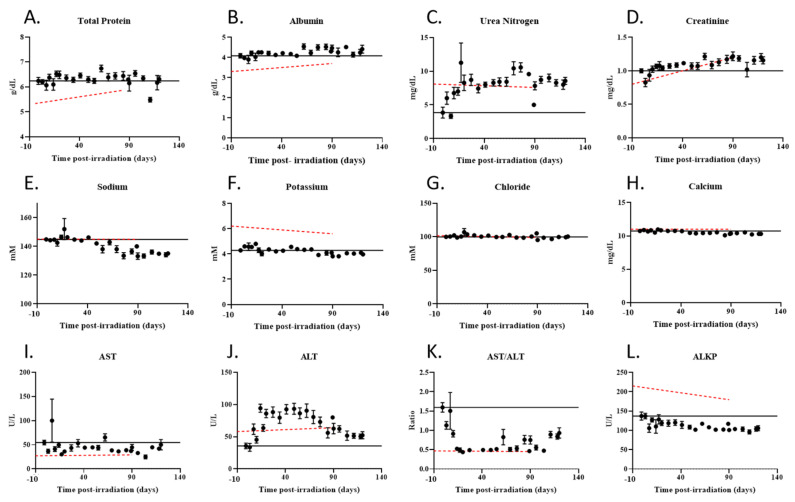
Longitudinal analysis of clinical chemistry parameters in the plasma of irradiated minipigs. (**A**–**L**) Reference values for each parameter were obtained from the vendor from non-irradiated, age-matched animals and are represented by the broken red line; the solid black line marks the pre-irradiation level of each parameter. Values are reported over time as averages +/− sem.

Clinical chemistry parameters were measured in irradiated animals and compared to reference data for age-matched Göttingen minipigs (Figure 5A–L). Some of the observed changes in clinical chemistry parameters followed the expected age-related trend. However, the changes in urea nitrogen (Figure 5C), sodium (Figure 5E), calcium (Figure 5H), aspartate transaminase (AST) (Figure 5I), and alanine transferase (ALT) (Figure 5J) levels differed from those associated with aging, indicating these changes were caused by radiation exposure. Plasma concentrations of urea nitrogen increased (Figure 5C) whereas sodium and calcium declined over time (Figure 5E,H) suggesting possible alterations in renal function leading to impairment of electrolyte reabsorption. Plasma IGF-1 levels in irradiated animals showed a weak but statistically significant positive association with sodium (R = 0.262, *p* = 0.003) and calcium (R = 0.263, *p* = 0.003) levels; sodium concentration in irradiated animals was also significantly associated (positive) with heart rate (*p* < 0.001) (Spearman’s rank test was conducted to assess associations). AST levels were reduced in irradiated animals (Figure 5I), likely suggestive of alterations in hepatic physiology, whereas ALT levels were increased (Figure 5J) leading to a sharp decline in the AST to ALT ratio (Figure 5K), indicative of radiation-induced liver damage.

**Figure 6:**
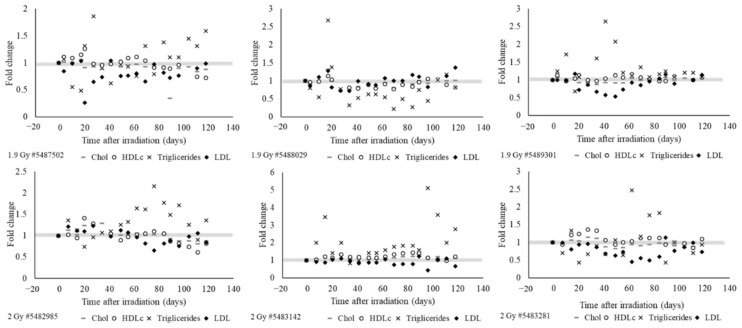
Longitudinal analysis of plasma lipid profiles in irradiated animals. Cholesterol, HDL, LDL, and triglyceride levels from individual animals were normalized to pre-irradiation levels and plotted over time. The grey box represents the pre-irradiation value measured for each parameter.

Development of dyslipidemia was observed in all irradiated animals (Figure 6). Irradiation mainly increased the triglyceride levels but suppressed the LDL levels. In all irradiated animals, HDL and cholesterol levels fluctuated inconsistently. Figure 6 depicts the lipid levels normalized to pre-irradiation values for 3 animals exposed to 1.9 Gy PBI and 3 animals exposed to 2.0 Gy PBI.

### 2.6. Radiation Blunted the Cardiac IGF-1 Signaling Pathway

IGF-1 ligand is known to bind to its respective receptor, IGF-1R, in cardiomyocytes and concomitantly activate two canonical pathways, the phosphatidylinositol-3 kinase/Akt pathway and the mitogen-activated protein kinase(MAPK) dependent pathway also known as extracellular signal-regulated kinase (Erk) pathway [30]. Activation of the MAPK/Erk pathway determines regulation of gene expression and modulates cardiac growth, mitogenesis, and differentiation whereas activation of the Akt pathway promotes survival and inhibition of apoptosis [51,52]. IGF-1 deficiency has been linked to dysregulated IGF-1 signaling and oxidative stress [21,53]. The expressional regulation of IGF-1 is an *in vivo* target of peroxisome proliferator-activated receptor (PPAR) alpha transcription factor [54]. The PPAR/IGF-1 pathway protects cardiomyocytes against apoptosis induced by ischemia and reperfusion or biomechanical stress. Activation of PPAR-alpha reduces triglyceride production and promotes plasma triglyceride clearance [54,55]. 

**Figure 7:**
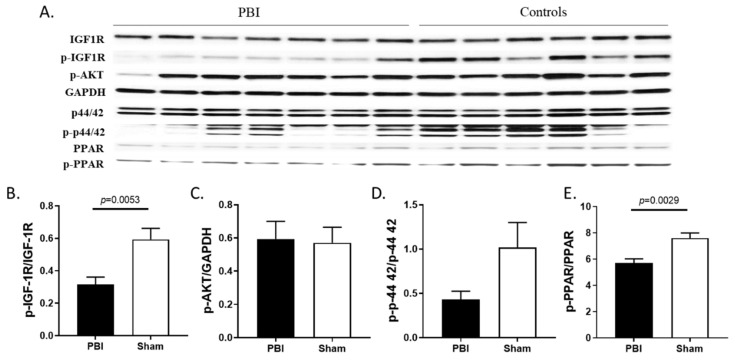
Western blot analysis of IGF-1 signaling pathway and PPAR activation in heart lysates of irradiated and control minipigs. (**A**) Evaluation of IGF-1R, Akt, p44/42 and PPAR phosphorylation in heart lysates of irradiated and control minipigs by SDS-PAGE and western blot. (**B**–**E**) Densitometric quantification was performed and values were normalized to respective loading control and presented as averages +/− sem.

Western blot analysis probing for the activated form of the IGF-1 receptor (phosphorylated on Tyrosine residues 1135/1136) showed significantly (*p* = 0.0053) decreased IGF-1 receptor (IGF-1R) activation in the hearts of irradiated animals [56] (Figure 7A,B). Western blot analysis showed Akt phosphorylation on Serine residue 473 remained relatively unchanged whereas phosphorylation of Erk (p44/42) was decreased, although not significantly (*p* = 0.058), with radiation exposure (Figure 7A,C,D). Since IGF-1 signaling is the upstream regulator of the effector kinases, Erk and Akt, these findings indicate IGF-1 signaling deficiency was associated with impairment of its downstream mediators. Consistent with inhibition of the IGF-1 pathway, cardiac PPAR-alpha activation marked by phosphorylation of its Serine 12 residue was significantly (*p* = 0.0029) downregulated following irradiation (Figure 7E).

### 2.7. The Role of IGF-1, TGF-beta, and PPAR-gamma in Differential Gene Expression as Seen by mRNA Sequencing

**Figure 8:**
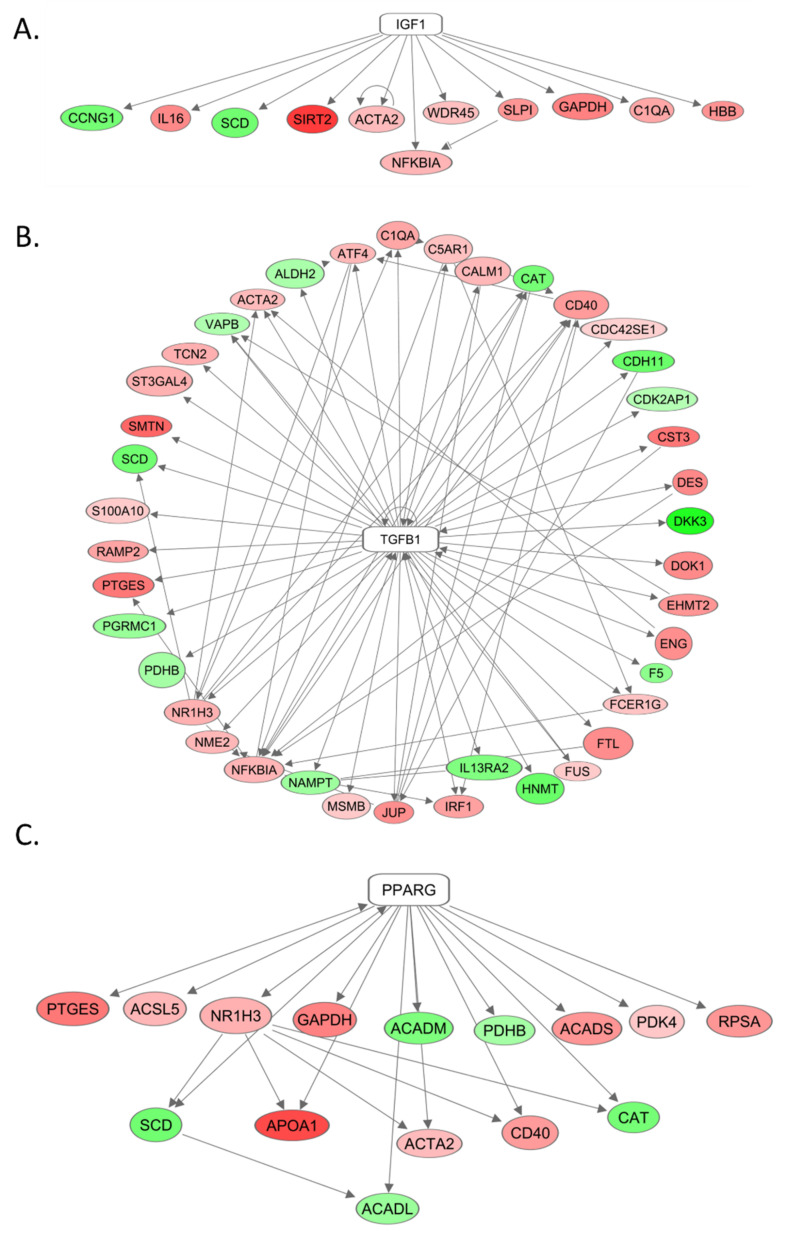
Schematic depiction of RNAseq analysis showing the key signaling nodes affected by irradiation in the hearts of minipigs. By mRNA sequencing and analysis, IGF-1 (**A**), TGF-beta (**B**), and PPAR-gamma (**C**) were identified as predicted upstream regulators in the heart of irradiated minipigs per z-score calculation. The oval and rectangular nodes represent candidate genes and corresponding regulators, respectively. The arrow-headed edges represent the relationships between the two nodes, and the red and green colors denote up- and down-regulated nodes, respectively.

To assess late effects of radiation exposure on gene expression alterations in heart tissue, we sequenced the total heart mRNA from irradiated animals and compared those results to that of control non-irradiated animals. Total mRNA sequencing analysis determined the existence of 122 upstream regulators of cardiac gene expression that met the hypergeometric cut-off *p* < 0.05 in the irradiated heart samples. Among them, IGF-1, TGF-beta, and PPAR-gamma are of particular interest. This is attributed to the z-score calculation outcomes that predicted IGF-1 as an activated upstream regulator (z score = 1.62) in irradiated heart tissues controlling 11 differentially expressed genes (DEG) that include 9 upregulated and 2 downregulated genes (Figure 8A). Likewise, TGF-beta emerged as a potentially activated upstream regulator (z score = 1.00) in the hearts of the irradiated minipigs, upregulating 27 genes and downregulating 13 genes (Figure 8B). PPAR-gamma is a potential upstream inhibitor (z score = 0.73) in irradiated animals as predicted by z-score algorithm. The family of genes regulated by PPAR-gamma included 10 upregulated and 5 downregulated genes (Figure 8C).

## 3. Discussion

Exposure to ionizing radiation not only triggers tissue injury through direct DNA damage but also will affect normal systemic activity and tissue signaling molecules including cytokines and hormones. This leads to the onset of pathologies associated with early or late effects of radiation exposure [57,58,59]. Patients with records of previous radiation therapy and survivors of accidental exposure to ionizing radiation manifest growth defects, endocrine disorders, metabolic imbalance, and cardiovascular defects years after exposure to harmful doses of radiation, which are commonly known as the late or delayed effects of radiation exposure [1,3,5].

Several animal models, primarily non-human primates and rodents, have been developed to study the delayed effects of acute radiation exposure [60,61,62,63,64,65,66,67,68,69,70]. These models mostly address the late effects in survivors following exposure to high doses of radiation. As such, they define the incidence, latency, severity, and dose- and time-dependent relationships for acute hematopoietic Acute Radiation Syndrome (H-ARS), gastrointestinal Acute Radiation Syndrome (GI-ARS), and lung injury with and without medical management [71,72]. In studies where partial body doses were delivered to animal models of delayed effects of acute radiation exposure in the range of 8 to 16 Gy, at later time points animals showed pathological manifestations in the intestine [73,74], kidney [75], and lungs [69,72,76,77]. Within nine years after exposure, non-human primates who received total body irradiation at doses between 6.5 and 8.4 Gy displayed higher prevalence of type 2 diabetes as well as lung, gastrointestinal, and cardiac injuries compared to age-matched, non-irradiated controls [10]. Rodents exposed to radiation doses of 7 Gy or higher developed hematopoietic dysfunction, lung and gastrointestinal damage [72,78].

While it is important to understand late health effects of radiation exposure at higher radiation doses as discussed above, in humans late health effects could occur at relatively lower doses of exposure. This is supported by epidemiological studies assessing health effects in the survivors of the atomic bombs; non-cancer, long term toxicities associated with radiation developed following exposure to doses as low as 0.5 Gy and included cardiotoxicity and respiratory, physiological, and hormonal changes [1,14,15]. Elevation in systolic and diastolic blood pressure, altered lipid profiles consisting of increased total cholesterol and triglycerides, and reduced high-density lipoprotein [1] are subclinical findings associated with radiation-dependent cardiovascular changes. Endocrine deficiencies have also been observed as early as three months after irradiation [5,18].

Here, we used a Göttingen minipig partial body irradiation (PBI) model to characterize clinical and subclinical changes and late health effects following relatively low but clinically relevant doses of radiation. Here, the Göttingen minipig PBI model included sparing of ~10% bone marrow containing the hematopoietic progenitors and tissues sufficient to reduce the extent of radiation-induced cytopenia. This resulted in accelerated recovery of blood elements and prevented development of systemic inflammation, thus reducing mortality at doses that could otherwise be lethal [49,50]. This allowed for extension of the study period to determine the associated late health effects up to 120 days post-irradiation. Additionally, the minipig offers an advantageous model for studying late effects as the anatomy and physiology of the pig closely resembles that of humans, with particular similarity of the immune system [46], metabolism [47], and cardiopulmonary systems [48].

In the current study, our data show radiation progressively induced circulating and tissue IGF-1 deficiency. In the heart, we observed inhibition of IGF-1 signaling, as seen in decreased IGF-1 receptor phosphorylation and activation of downstream targets or effectors of IGF-1, Erk (p 44/42), a well-documented mediator of heart growth [52], and PPAR-alpha. mRNA sequencing and analysis identified IGF-1 as a predicted activated upstream regulator of cardiac gene expression, linking it to the differential gene expression seen following irradiation. TGF-beta and PPAR-gamma were also shown by mRNA sequencing and analysis to influence radiation-induced gene expression changes. Identifying IGF-1, TGF-beta, and PPAR-gamma as upstream regulators of delayed effects of radiation injury could have significant clinical potential in preventing late health effects of radiation exposure. This is owing to the fact that a large pool of genes and their biological functions can be systematically controlled via targeting each one of these upstream signaling nodes.

The IGF-1 deficit was not sufficient to induce systemic inflammation, atherosclerosis, or alterations in blood pressure, likely due to the relatively short observation time. IGF-1 deficiency was associated with reduction in the rate of body weight gain, alteration in cardiac, renal, and hepatic function, and altered lipid metabolism. Liver function and hepatic architecture were altered following irradiation as determined by presence of fibrosis and altered ALT and AST to ALT ratios, indicators of liver damage. These results are consistent with the finding that IGF-1 signaling impairment is involved in chronic liver disease development [79]. Radiation exposure induced additional cardiac effects including arrhythmia, decreased heart rate, increased collagen deposition as seen in cardiac histopathology, and development of cardiac fibrosis.

Our data are aligned with the documented role of IGF-1 deficiency in the pathophysiology of both cardiovascular conditions and metabolic syndrome [18,19,20,21,22,23,24,25,26,27,28]. IGF-1 stimulates growth and development in vertebrates and maintains organ structure and homeostasis [80,81,82]. Consistent evidence from in vitro and in vivo studies have demonstrated the association between IGF-1 deficit and dysregulation of the GH/IGF-1 signaling pathways in dwarfism, arrhythmia, reduced heart contractility, inhibition of renal electrolyte reabsorption, and altered lipid metabolism, among other pathologies [18,19,20,36,53]. IGF-1 deficiency is also associated with liver and heart fibrosis [53,79]. The administration of IGF-1 has been shown to preclude fibrosis and ameliorate liver function in a rat model of liver cirrhosis [83] and recovery of heart functions and inhibit myocardial fibrosis in a mouse model of dilated cardiomyopathies [84].

### Conclusions and Limitations

In conclusion, we demonstrated that the Göttingen minipig is a suitable model for studying the late effects of radiation exposure on the endocrine system and the major organs. This study represents the initial characterization of the model; it has several limitations including the low number of animals, the relatively short observation period compared to animal life span, and the lack of in-house controls. However, our work highlights the minipig as a model for studying effects of clinically relevant, relatively low doses of radiation and potentiates further investigation into the role of growth hormones in the development of the late effects of radiation exposure. Among the main strengths presented here are the similarities between late effects of radiation in the model and known consequences of radiation exposure in humans, including IGF-1 deficiency, growth delays, dyslipidemia, and heart, kidney, and liver complications. Given the central physiological role of IGF-1 in controlling organ function and structure, radiation-induced IGF-1 deficiency is of particular interest. Radiation-induced IGF-1 signaling perturbations offer an explanation to several of the late effects observed in humans including the systemically coordinated failure of the internal organs and development of diabetes, obesity, metabolic syndromes, and cardiovascular diseases.

## 4. Materials and Methods

### 4.1. Animal Care and Irradiation

Animal husbandry was maintained as previously described [21,43]. Briefly, eight male Göttingen minipigs (3 months old) were purchased from Marshall Bioresources Inc. then housed in the Armed Forces Radiobiology Research Institute (AFRRI) Veterinary Science Department (VSD); where they were acclimatized for 2 weeks prior to irradiation. Animals were provided free access to water and pig chow (~175 g) twice daily as per animal vendor’s recommendations. Animal rooms were maintained at 61–81 °F with 30–70% relative humidity and 12 h light–dark cycles. Minipigs were singly housed to allow individual assessment of feed consumption and fecal/urine/blood production. Although singly housed, visual, olfactory, and auditory contact with other minipigs was allowed to support their highly social nature.

Prior to irradiation, animals were anesthetized with a mixture of Telazol (2 mg/kg) and xylazine (1 mg/kg) (single intramuscular injection) and were exposed to partial body irradiation (PBI), sparing the tibia of the lower limbs (10% bone marrow sparing) using LINAC Elekta Integrity. Additional minipigs (6–7 months old) served as controls for western blot analysis and histology. Body weights and clinical chemistry control values from age-matched, non-irradiated, male Göttingen minipigs were obtained from Marshall Bioresources. Research was conducted under an animal use protocol approved (Protocol number 2015-10-008) by the AFRRI Institutional Animal Care and Use Committee on December 11, 2015 in an AAALAC-accredited (Association for Assessment and Accreditation of Laboratory Animal Care) facility in compliance with the Animal Welfare Act and other federal statutes and regulations relating to animals and experiments involving animals. This work additionally adheres to principles stated in the Guide for the Care and Use of Laboratory Animals, National Research Council (NRC) Publication, 2011 edition.

Minipigs were irradiated between 8 am and 12 pm with 2 Gy (*n* = 4) or 1.9 Gy (*n* = 4). Anesthetized animals were irradiated in the prone position directly on the table surface in the PA/AP mode with 4 MV photon beam (mean energy 1.2 MeV) with a dose rate of ~0.6 Gy/min. Half of the dose (PA) was given at the gantry angle of 0° and the second half (AP) was given at the gantry angle of 180°. The dose was prescribed to the midplane of each animal. To spare the tibia of the lower limbs, the animal was positioned with respect to the edge of the light field under a collimator angle of 45°. Although the position of the animal remained the same for both fractions of irradiation, the collimator was rotated by 180° for the AP fraction in order to account for any possible asymmetry of the legs. 

One animal exposed to 2 Gy became moribund from hematopoietic Acute Radiation Syndrome and was euthanized before the completion of the observation period. The remaining animals were followed for 120 days post-irradiation (post-IR) and were provided minimum preventive supportive care (anti-inflammatory, antibiotic, and nutritional support) throughout the course of the study. The results of blood cell counts and plasma and tissue analyses from the 1.9 Gy dose group and the 2 Gy dose group were combined.

### 4.2. Blood Sampling and Plasma and Tissue Collection

Blood samples were obtained longitudinally on days −7 (pre-irradiation (pre-IR)), 1, 3, 7, 10, 14, 17, 20, 23, 27, 30, and twice a week thereafter until the end of the study (day 120). The day of irradiation was considered day 0. Blood was collected from peripheral veins under isoflurane anesthesia. Complete blood cell counts were determined in whole blood collected with EDTA. Plasma was separated from sodium heparinized blood by centrifugation at 1000× *g* for 10 min. Clinical chemistry parameters were analyzed in plasma. The remainder of the plasma was stored at −80 °C for biomarker analysis by ELISA. At the end of the study, animals were anesthetized (5–2% isoflurane; intramuscular (IM) injection of Telazol) and euthanized by IV administration of sodium pentobarbital (Euthasol^®^). Organs were excised and tissues were either frozen and stored at −80 °C for later use or fixed in 10% zinc-buffered formalin for histological analysis.

### 4.3. Histology

Tissues were fixed in neutral buffered zinc-formalin for 48 h then transferred to 70% ethanol. Trimmed tissues were embedded in paraffin and 8 µm sections were cut from the blocks, deparaffinized, and stained with hematoxylin and eosin (H&E); heart (left ventricle), lung (right diaphragmatic lobe), liver, and kidney (left) sections were stained with Masson’s Trichrome. All slides were analyzed by a board certified pathologist.

### 4.4. Preparation of Cytosolic and Membrane Fractions and Immunoblotting 

Frozen heart tissues were minced with surgical scissors and homogenized using Tissue Tearors (BioSpec Products, Inc., Bartlesville, OK, USA; Models 985370, 985370-395); membrane and cytosolic protein fractions were extracted using the Mem-PER Plus kit (Thermo Fisher, Waltham, MA, USA; PI89842). Protein was quantified by Bicinchoninic acid assay (BCA) (Thermo Fisher, Waltham, MA, USA; PI23225). Lithium dodecyl sulfate (LDS) buffer (Thermo Fisher, Waltham, MA, USA; NP0007) and reducing agent (Thermo Fisher, Waltham, MA, USA; NP0009) were added to a 1× concentration and boiled for 10 min at 80 °C. Protein was separated in 4–15% Tris Glycine (5671084) or 4–12% BisTris (3450124) (Bio-Rad, Hercules, CA, USA) gels by SDS-PAGE and wet-transferred to polyvinylidene fluoride (PVDF) membranes using 100 V for 1 h.

Blots were washed with 1X Tris-buffered saline (TBS) with 0.05% Tween20 and blocked with StartingBlock TBS buffer (Thermo Fisher, Waltham, MA, USA; EN37543) for 1 h at room temperature. Primary and secondary antibodies were diluted in the same blocking buffer. Membranes were incubated with primary antibodies overnight at 4 °C. Antibodies were used at the following dilutions: 1:500 p-AKT (Cell Signaling, Danvers, MA, USA; 9271), 1:1000 p-PPAR-alpha (ab3484) and 1:1000 PPAR-alpha (ab126285) (abcam, Cambridge, UK), and 1:1000 TGF-beta (Invitrogen, Carlsbad, CA, USA; MA5-15065). Secondary antibodies, Goat anti-Rabbit IgG (H+L), HRP (Horseradish Peroxidase), Superclonal (A27036) and Goat anti-Mouse IgG (H+L), HRP, Superclonal (A28177) (Invitrogen, Carlsbad, CA, USA), were diluted 1:10,000 and incubated with membranes for 1 h at room temperature. Membranes were incubated with Enhanced Chemiluminescence ECL substrate (Thermo Fisher, Waltham, MA, USA; PI34577) for 5 min at room temperature, exposed to x-ray films (Thermo Fisher, Waltham, MA, USA; PI34090), and developed. Blots were also imaged using the Bio-RAD ChemiDoc MP Imaging System (Hercules, CA, USA).

A subset of blots were processed using the Western Breeze chemiluminescent kit (Thermo Fisher, Waltham, MA, USA; WB7106; WB7104). Blots were washed and blocked for 1 h at room temperature with provided buffers. Primary antibodies were diluted in provided diluent and incubated with membranes overnight at 4 °C. Antibodies were used at the following dilutions: 1:500 IGF-1R (9750) and 1:500 p-IGF-1R (3024) (Cell Signaling, Danvers, MA, USA). Membranes were incubated with provided secondary antibody for 1 h at room temperature then incubated with chemiluminescent substrate for 5 min at room temperature in the absence of light. Blots were developed and imaged as described previously.

PonceauS (Sigma Aldrich, St. Louis, MO, USA; P7170-1L) and GAPDH (Cell Signaling, Danvers, MA, USA; 2118L) were used as loading controls for membrane and cytosolic blots, respectively. GAPDH was diluted 1:40,000 in StartingBlock TBS and incubated with membranes for 1 h at room temperature. Goat anti-Rabbit IgG (H+L), HRP, Superclonal (A27036) secondary was diluted 1:20,000 in the same buffer and incubated for 1 h at room temperature. Blots were developed and imaged as described previously. Protein was quantified by densitometry with Image Lab 5.2.1 (Bio-Rad, Hercules, CA, USA).

### 4.5. Determination of Plasma IGF-1 and C-Reactive Protein (CRP)

IGF-1 and CRP were analyzed in plasma by ELISA. Previously frozen plasma was thawed at room temperature and purified by centrifugation at 9500× *g* at 4 °C for 30 min. Sodium heparinized plasma was diluted 1:100 for IGF-1 assays (Biomatik, Kitchener, Ontario, Canada; EKU04969) and 1:2000 for CRP assays (Genway Biotech, San Diego, CA, USA; GWB-699A6F). Both assays were performed according to the manufacturers’ protocols.

### 4.6. Collagen Staining and Measurement

To quantify collagen deposition in the heart, samples were prepared as described previously [85]. Briefly, 5 µm tissue sections were rehydrated and incubated with Sirius Red supplemented with Fast Green. ScanScope CS2 slide scanner was used to scan sections; subsequently, scans were analyzed using ImageScope 12 software (Leica Biosystems, Wetzlar, Germany). The relative tissue area of collagen was calculated as the red-stained area expressed as a percentage of the total tissue area of each section.

### 4.7. Sample Preparation and mRNA Sequencing

Frozen heart tissue was cryogenically ground to a powder using Cryomill (Retsch GmbH, Haan, Germany). TRIzol reagent (Invitrogen, Thermo Fisher Scientific, Wilmington, MA, USA) was added to ≈10 mg of tissue and the sample was homogenized further using the Precellys Evolution Homogenizer and Cryolys Evolution (Bertin Technologies SAS, Montigny-le-Bretonneux, France). RNA was extracted using a combination of two procedures, Invitrogen’s TRIzol RNA extraction methods and miRNeasy mini kit (QIAGEN Inc., Germantown, MD, USA) total RNA purification procedure. TRIzol reagent was used to facilitate lysis of the tissue, inhibit RNases, and also, with the addition of chloroform, separate cellular DNA and proteins from the RNA. RNA is contained in the upper aqueous phase, while DNA and protein is contained in the lower interphase and organic phase, respectively. The aqueous phase was extracted and ethanol was added to provide optimal binding conditions for all RNA molecules. The sample was then applied to the RNeasy mini spin column per manufacturer’s instructions. Total RNA was eluted in RNase-free water and quality control checks were performed using NanoDrop spectrophotometer (Thermo Fisher Scientific, Wilmington, MA, USA) and Agilent 4200 TapeStation (Agilent Technologies, Santa Clara, CA, USA).

The sequencing library preparation was done using 500 ng of total RNA input with the TruSeq Stranded mRNA Sample Preparation Kit (Illumina Inc, San Diego, CA, USA) following the manufacturer’s procedures. In brief, for fragmentation, total RNA was exposed to divalent cations under elevated temperature and first strand cDNA was synthesized from cleaved RNA fragments using reverse transcriptase SuperScript II (ThermoFisher, Waltham, MA, USA). Strand selection was done by replacing deoxythymidine triphosphate (dTTP) with deoxyuridine triphosphate (dUTP) then DNA Polymerase I and RNase H (Illumina Inc, San Diego, CA, USA) were used for second strand cDNA synthesis. A tail was added to the 3’ ends of the blunt fragments. Unique indexing adapters were ligated to the ends of the ds cDNA fragments. Libraries were finally enriched in preparation for sequencing after quantification with real-time quantitative PCR (qPCR; Kapa Biosystems, Wilmington, MA, USA). Size and quality of the libraries were determined using the High Sensitivity D1000 ScreenTape Assay with the 2200 TapeStation (Agilent Technologies, Santa Clara, CA, USA). Paired end sequencing was then performed on Illumina HiSeq4000 sequencer using the HiSeq 3000/4000 SBS Kit (300-cycle) (Illumina Inc, San Diego, CA, USA,) loading four indexed libraries per lane.

mRNA raw unaligned reads were trimmed of Illumina adapters using Trimmomatic v0.38 and aligned to the Sus scrofa genome v11.1 from Ensembl. mRNA reads were aligned with the Spliced Transcripts Alignment to a Reference (STAR) aligner v2.6.0 (Cold Spring Harbor Laboratory, Cold Spring Harbor, NY, USA and Pacific Biosciences, Menlo Park, CA, USA). Genes were filtered to include only genes with a counts per million (CPM) greater than 0 across 20 samples and were normalized with the weighted trimmed mean of M-values method using the edgeR package v3.24.3. Differential expression was calculated by fitting a negative binomial generalized log-linear model to the read counts for each gene using the limma package v3.38.3 (R Foundation for Statistical Computing, Vienna, Austria).

mRNA sequencing was used to determine upstream regulators, defined as any molecular entity that is known to affect the expression of a single gene or a family of genes via direct or indirect causal association (PMID: 24336805). Upstream Regulator Analysis (URA) in the Ingenuity Pathway Analysis (IPA) platform was adapted from the Connectivity Map tool (PMID: 17008526), where the relationships among the genes were mapped using database developed by literature curation.

### 4.8. Statistical Analysis

Microsoft Excel (Microsoft, Redmond, WA, USA) and GraphPad Prism 8 (Scientific Software, San Diego, CA, USA) were used to calculate standard error of the mean (sem) for all values shown. GraphPad Prism 8 (Scientific Software, San Diego, CA, USA) was used to calculate statistical differences between irradiated and non-irradiated groups for area of collagen deposition and densitometry. Correlations were determined by Spearman’s rank test. For mRNA sequencing, moderate t-test was performed to find differentially expressed genes (DEG) between the irradiated and non-irradiated baseline at *p* < 0.05. These DEGs were seeded in IPA to determine the significant upstream regulators pertinent to the cardiac functions. Hypergeometric test was used to define the significance cut-off at *p* < 0.05.

## Figures and Tables

**Table 1:** Histological findings in Masson’s Trichrome- and H&E-stained slides from the minipig-PBI model. Findings were scored by a board certified pathologist and summarized based on extent of lesion observed.

Dose (Gy)	Heart, Left Ventricle and Atrium	Lung, Right Diaphragmatic Lobe	Left Kidney
2	arterial pigmentation	hemorrhage, mild fibrosis	nephropathy, mild fibrosis
2	arterial pigmentation	minimal fibrosis	nephropathy, mild fibrosis
2	no visible lesions	no visible lesions	mild fibrosis
1.9	arterial pigmentation	edema, congestion	mild fibrosis
1.9	no visible lesions	minimal fibrosis	minimal fibrosis
1.9	no visible lesions	no visible lesions	mild fibrosis
1.9	no visible lesions	no visible lesions	minimal fibrosis

## Abbreviations

IGF-1	Insulin-like growth factor-1
GH	Growth hormone
PBI	Partial body irradiation
CRP	C-reactive protein
MV	Megavolt
LINAC	Linear accelerator
TGF-beta	Transforming growth factor beta
AST	Aspartate transaminase
ALT	Alanine transferase
HDL	High-density lipoprotein
LDL	Low-density lipoprotein
IGF-1R	Insulin-like growth factor-1 receptor
MAPK	MAP kinase
PPAR	Peroxisome proliferator-activated receptor
H-ARS	Hematopoietic Acute Radiation Syndrome,
GI-ARS	Gastrointestinal Acute Radiation Syndrome
mRNA	Messenger Ribonucleic Acid
URA	Upstream Regulator Analysis
IPA	Ingenuity Pathway Analysis
DEG	Differentially expressed genes
sem	Standard Error of Mean
